# Trends in SARS-CoV-2-related pediatric hospitalizations reported to the Canadian Nosocomial Infection Surveillance Program, March 2020 to December 2022

**DOI:** 10.1017/ash.2024.427

**Published:** 2024-10-17

**Authors:** Diane Lee, Erin McGill, Linda Pelude, Robyn Mitchell, Jeannette L. Comeau, Charles Frenette, Bonita E Lee, Marie-Astrid Lefebvre, Jocelyn A. Srigley, Nisha Thampi

**Affiliations:** 1 Centre for Communicable Diseases and Infection Control, Public Health Agency of Canada, Ottawa, ON, Canada; 2 Department of Pediatrics, Dalhousie University, Halifax, NS, Canada; 3 Division of Infectious Diseases, Department of Medicine, McGill University Health Centre, Montréal, QC, Canada; 4 Department of Pediatrics, University of Alberta, Stollery Children’s Hospital, Edmonton, AB, Canada; 5 Montreal Children’s Hospital, McGill University Health Centre, Montréal, QC, Canada; 6 Department of Pathology and Laboratory Medicine, BC Children’s and BC Women’s Hospitals, Vancouver, BC, Canada; 7 Department of Pediatrics, Children’s Hospital of Eastern Ontario, Ottawa, ON, Canada

## Abstract

**Objective:**

This study describes trends in COVID-19 hospitalizations and healthcare-associated (HA) COVID-19 in Canada among pediatric (age <18 years) patients during pre-Omicron and Omicron-dominant periods.

**Design:**

Prospective surveillance for COVID-19 infection

**Setting:**

The Canadian Nosocomial Infection Surveillance Program is a sentinel surveillance system with 45 hospitals providing COVID-19 data on pediatric patients, including all 13 pediatric tertiary care facilities in Canada.

**Patients:**

Pediatric patients hospitalized with laboratory-confirmed COVID-19 at a participating hospital between March 1, 2020, and December 31, 2022.

**Methods:**

Analyzed case-level data on pediatric patients with COVID-19, including demographics, acquisition source, and outcomes.

**Results:**

Among 5,143 pediatric cases, the majority (81%) were reported during the Omicron-dominant period (beginning December 26, 2021). However, a lower proportion required intensive care during the Omicron wave (11% vs 14%, *P* < 0.05); no difference in mortality was observed. Of those patients admitted to hospital due to COVID-19 (n = 2,700), 45% had at least one pre-existing comorbidity. The majority (90%) of patients with HA-COVID-19 were reported during the Omicron period. There was no difference in mortality between patients with HA and community-associated (CA) infection, although a greater proportion of CA infections led to intensive care unit admission (6% vs 13%, *P* < 0.01).

**Conclusions:**

Surveillance findings indicate that both HA- and CA-COVID-19 hospitalizations in Canada increased among pediatric patients following the emergence of the Omicron variant, although disease severity decreased. Pre-existing health conditions were common among pediatric patients hospitalized with COVID-19, highlighting the importance of preventing severe illness in this sub-population.

## Introduction

Early in the COVID-19 pandemic, illness severity and outcomes among pediatric patients were of particular interest due to historical high burdens of respiratory viral infections among this population and observed severe illness for influenza and respiratory syncytial virus at both ends of the age spectrum. In addition, the effects of COVID-19 among pregnant and neonatal populations were unknown.^1–3^

The effect of COVID-19 on the pediatric population has evolved over time. In 2020, SARS-CoV-2 infection was reportedly milder in children compared to adults and some children were asymptomatic.^4–8^ Children accounted for a small proportion of diagnosed COVID-19 cases (approximately 4% in Canada) and deaths were extremely rare.^4,7–11^ Due to these early trends, SARS-CoV-2 vaccine rollout was targeted at older adults with a later and staggered rollout for different pediatric age groups in Canada.

With the emergence of the highly transmissible Omicron variant, there was an increase in hospitalizations, including among the pediatric population.^12^ Research suggested that young children were more susceptible to complications from upper respiratory infections and Omicron more readily infects cells in the upper airways.^13,14^

This study describes the trends in pediatric cases (age < 18 years) who had laboratory-confirmed COVID-19 on admission or who acquired COVID-19 during hospitalization across Canada between March 1, 2020, and December 31, 2022, covering pre-Omicron and Omicron-dominant periods, and comparing patients with healthcare-associated (HA)-COVID-19 and community-associated (CA)-COVID-19. We also examine differences in pre-existing comorbidities across age groups.

## Methods

### Data sources and data collection

The Canadian Nosocomial Infection Surveillance Program (CNISP) is a sentinel surveillance system that collects epidemiologic and linked microbiology data from 106 acute care hospitals across 10 provinces and one territory, representing 37% of acute care beds in Canada. The surveillance system is a collaboration between the Public Health Agency of Canada, the National Microbiology Laboratory, the Association of Medical Microbiology and Infectious Disease Canada, and sentinel hospitals. With the emergence of SARS-CoV-2, viral respiratory infection (VRI) surveillance for COVID-19-associated hospitalizations began in March 2020 and collected information for both CA- and HA-COVID-19 cases. Currently, 93 CNISP acute care hospitals participating in VRI surveillance from 10 provinces and one territory submit patient-level questionnaire data on a quarterly basis. Of the 93 CNISP hospitals participating in VRI surveillance, 45 contributed data on pediatric patients and were included in the analysis, including all 13 pediatric tertiary care centers in Canada.

The surveillance definition for this study includes hospitalized pediatric patients with laboratory-confirmed COVID-19 (positive SARS-CoV-2 polymerase chain reaction (PCR) while in hospital or within 14 days prior to hospital admission) between March 1, 2020, and December 31, 2022. The COVID-19 patient questionnaire collects information on demographics, acquisition source, reason for admission, severity (including intensive care unit (ICU) admission, mechanical ventilation, and extracorporeal membrane oxygenation, death), risk factors, vaccination status, treatment and 30-day outcomes. Data were collected through review of patient medical records by trained infection control professionals and entered into a secure web-based platform hosted on the Canadian Network for Public Health Intelligence.

### Definitions

The following definitions were applied to all cases submitted to the CNISP VRI system during the study period:^15^

#### COVID-19 case

- Positive SARS-CoV-2 PCR while in hospital or within 14 days prior to hospital admission.

#### Healthcare-associated (HA) COVID-19 case

- Symptom onset or positive test result 7 calendar days or more after admission to the reporting hospital and using best clinical judgment OR
- Symptom onset less than 7 days after admission but known epidemiological link to a positive case OR
- Readmission with a positive test less than 7 calendar days after discharge from hospital OR
- Any patient identified with COVID-19 is not acquired within the reporting hospital but is thought to be associated with another healthcare facility (e.g., another acute care facility).

#### Community-associated (CA) COVID-19 case

- No exposure to healthcare that would have resulted in this infection (using best clinical judgment) and does not meet the criteria for an HA infection.

Specific criteria were used to help determine if a patient’s admission was related to COVID-19 (i.e. patient was admitted due to COVID-19 or COVID-19 contributed to their admission). Data collectors were instructed to use their best clinical judgment when applying the criteria and/or other available clinical information to determine if the admission is COVID-19 related or not.^15^

Pediatric patients who met the CNISP COVID-19 case definitions were included in the analyses, including both patients admitted due to COVID-19-related illness and SARS-CoV-2-positive patients hospitalized for other reasons. However, diagnoses of Multisystem Inflammatory Syndrome in Children among these patients were not captured.

Wave cut points were determined by the CNISP VRI Working Group using visual assessment of the data to identify when increases and decreases were observed in the proportion of COVID-19 hospitalizations reported to CNISP. Seven waves were defined as wave 1 (March 1–August 31, 2020), wave 2 (September 1, 2020–February 28, 2021), wave 3 (March 1–June 30, 2021), wave 4 (July 1–December 25, 2021), wave 5 (December 26, 2021–March 19, 2022), wave 6 (March 20–May 28, 2022) and wave 7 (May 29–December 31, 2022).^16^ Waves 1 through 4 were in the pre-Omicron period, while waves 5 through 7 were in the Omicron-dominant period.

In Canada, pediatric vaccine rollout was staggered as Health Canada approved the COVID-19 vaccine for different pediatric age groups at separate times. The COVID-19 vaccine was approved for individuals aged 12–17 and individuals aged 5–11 years on May 5, 2021, and November 19, 2021, respectively.^17,18^ The vaccine was not approved for individuals aged 6 months to 5 years until July 2022.^19^

### Statistical analysis

Descriptive statistics were computed to compare patient characteristics during pre-Omicron and Omicron-dominant periods, and to compare HA- and CA-COVID-19. Age categories used in analyses were determined using vaccine eligibility groups for pediatric populations in Canada.^19^ Proportions were calculated for different variables of interest within the pediatric population meeting the COVID-19 case definition. Differences in proportions were calculated using Chi-squared test or Fisher’s exact test, and medians were compared using Kruskal-Wallis rank sum tests. For all statistical tests, *P* < 0.05 was considered statistically significant. Missing and incomplete data for individual variables were excluded from analyses, therefore denominators varied across variables being compared. All analyses were conducted using R version 4.0.5.^20^

## Results

**Figure 1.**
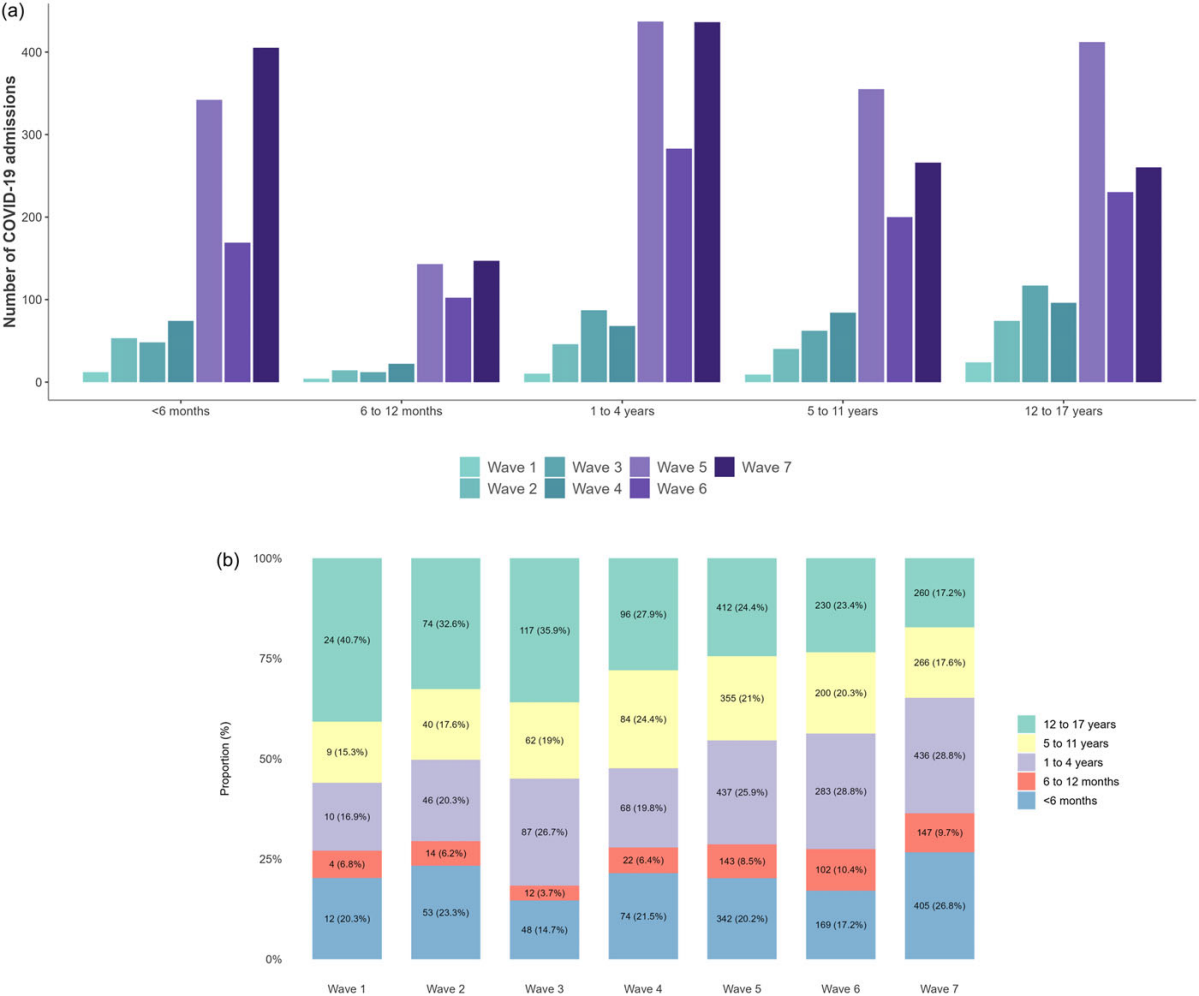
a Number of pediatric patients hospitalized with laboratory-confirmed COVID-19 by age group and wave. b. Proportion of pediatric patients hospitalized with laboratory-confirmed COVID-19 by age group and wave. Seven waves were defined as wave 1 (March 1–August 31, 2020), wave 2 (September 1, 2020–February 28, 2021), wave 3 (March 1–June 30, 2021), wave 4 (July 1–December 25, 2021), wave 5 (December 26, 2021–March 19, 2022), wave 6 (March 20–May 28, 2022) and wave 7 (May 29–December 31, 2022). Waves 1 through 4 were in the pre-Omicron period, while waves 5 through 7 were in the Omicron-dominant period.

A total of 5,143 pediatric patients from 45 hospitals were reported to have laboratory-confirmed COVID-19 between March 1, 2020, and December 31, 2022, with 4,187 (81%) reported during the Omicron-dominant period (Figure 1a). Table 1 presents characteristics of hospitalized pediatric patients during pre-Omicron and Omicron-dominant periods. Compared to pre-Omicron, the median age of pediatric patients with COVID-19 during the Omicron period decreased from 5 years to 3 years (*P* < 0.001), and a higher proportion of patients under five years of age was observed (59% vs 47%, *P* < 0.001) (Figure 1b). The proportion of HA vs. CA cases in pediatric cases almost doubled during the Omicron period (5.9% vs 3.2%, *P* < 0.001) and the proportion of patients whose admission was attributable to their COVID-19 illness increased from 47% to 54% (*P* < 0.001). Proportions of patients treated with antivirals increased during the Omicron period (7.1% vs 5.2%, *P* < 0.05), while treatment using steroids did not significantly differ across time periods. Remdesivir use was only captured during the Omicron period, during which 4.2% (119/2,867) of all pediatric COVID-19 patients were treated using remdesivir. Among patients who had been discharged within 30 days of positive COVID-19 test, length of hospital stay decreased from 3 days pre-Omicron to 2 days during Omicron (*P* < 0.001). Among patients eligible for vaccination at the time of COVID-19-related hospitalization, 79% had not received a COVID-19 vaccine during pre-Omicron versus 49% during Omicron (*P* < 0.001). Among patients with COVID-19-related ICU admission during the pre-Omicron period, 96% had not received a COVID-19 vaccine compared to 72% during Omicron (*P* < 0.001). A lower proportion of patients required intensive care due to COVID-19 during Omicron (11% vs 14%, *P* < 0.05), though no difference in mortality was observed between periods (1.0% (10/956) pre-Omicron vs 1.0% (28/2,938) during Omicron). Although pediatric deaths were rare, 90% were attributable to COVID-19 (Table 1).

**Table 1.** Characteristics of hospitalized pediatric patients with COVID-19 during pre-Omicron and Omicron-dominant waves, March 2020–December 2022

Characteristic	Pre-Omicron (Mar 2020–Dec 2021) n = 956^a^	Omicron (Jan 2022–Dec 2022) n = 4,187^a^	*P* value^b^
**Age (years)**			<0.001
Median (IQR)	5.0 (1.0, 13.0)	3.0 (0.0, 10.0)	
Range	0.0, 17.0	0.0, 17.0	
**Age category**			
<6 months	187/956 (20%)	916/4,187 (22%)	0.12
6–12 months	52/956 (5.4%)	392/4,187 (9.4%)	<0.001
1–4 years	211/956 (22%)	1,156/4,187 (28%)	<0.001
5–11 years	195/956 (20%)	821/4,187 (20%)	0.58
12–17 years	311/956 (33%)	902/4,187 (22%)	<0.001
**Sex**			0.20
Male	534/949 (56%)	2,248/4,164 (54%)	
Female	415/949 (44%)	1,916/4,164 (46%)	
**Reason for admission** ^c^			<0.001
Hospitalized due to COVID-19	447/943 (47%)	2,253/4,179 (54%)	
Hospitalized for other reasons	496/943 (53%)	1,926/4,179 (46%)	
**Acquisition source**			<0.001
Community-associated	859/887 (97%)	3,921/4,165 (94%)	
Healthcare-associated	28/887 (3.2%)	244/4,165 (5.9%)	
**Length of stay** (days)	3.0 (2.0, 6.0)	2.0 (1.0, 5.0)	<0.001
**Number of COVID-19 vaccine doses received** ^d^			
0	83/105 (79%)	419/860 (49%)	<0.001
1	12/105 (11%)	119/860 (14%)	0.50
2	10/105 (9.5%)	287/860 (33%)	<0.001
3+	0/105 (0%)	35/860 (4.1%)	0.026
**Pre-existing comorbidity**	413/938 (44%)	1,337/2,876 (46%)	0.19
**Symptomatic**	751/922 (81%)	2,647/2,870 (92%)	<0.001
**Receipt of antiviral**	48/931 (5.2%)	204/2,867 (7.1%)	0.037
**Receipt of steroids**	242/872 (28%)	829/2,873 (29%)	0.53
**ICU admission (all-cause)**	185/952 (19%)	418/2,910 (14%)	<0.001
ICU admission attributable to COVID-19	134/952 (14%)	331/2,910 (11%)	0.026
**Mechanical ventilation**	73/943 (7.7%)	202/2,890 (7.0%)	0.44
**Extracorporeal membrane oxygenation**	3/947 (0.3%)	4/2,958 (0.1%)	0.37
**30-day outcome**			
Patient survived and discharged	880/956 (92%)	2,718/2,938 (93%)	0.64
Patient alive, still in hospital	33/956 (3.5%)	111/2,938 (3.8%)	0.64
Patient survived and transferred	32/956 (3.3%)	65/2,938 (2.2%)	0.051
Patient died (all-cause)	10/956 (1.0%)	28/2,938 (1.0%)	0.80
Death attributable to COVID-19	9/10 (90%)	19/22 (86%)	>0.99

IQR, interquartile range; ICU, intensive care unit.

a n/N (%).

b Wilcoxon rank sum test; Pearson’s Chi-squared test; Fisher’s exact test.

c Diagnoses of Multisystem Inflammatory Syndrome in Children (MIS-C) among these patients were not captured.

d Patients ineligible for vaccination at time of positive SARS-CoV-2 test were excluded.

**Figure 2.**
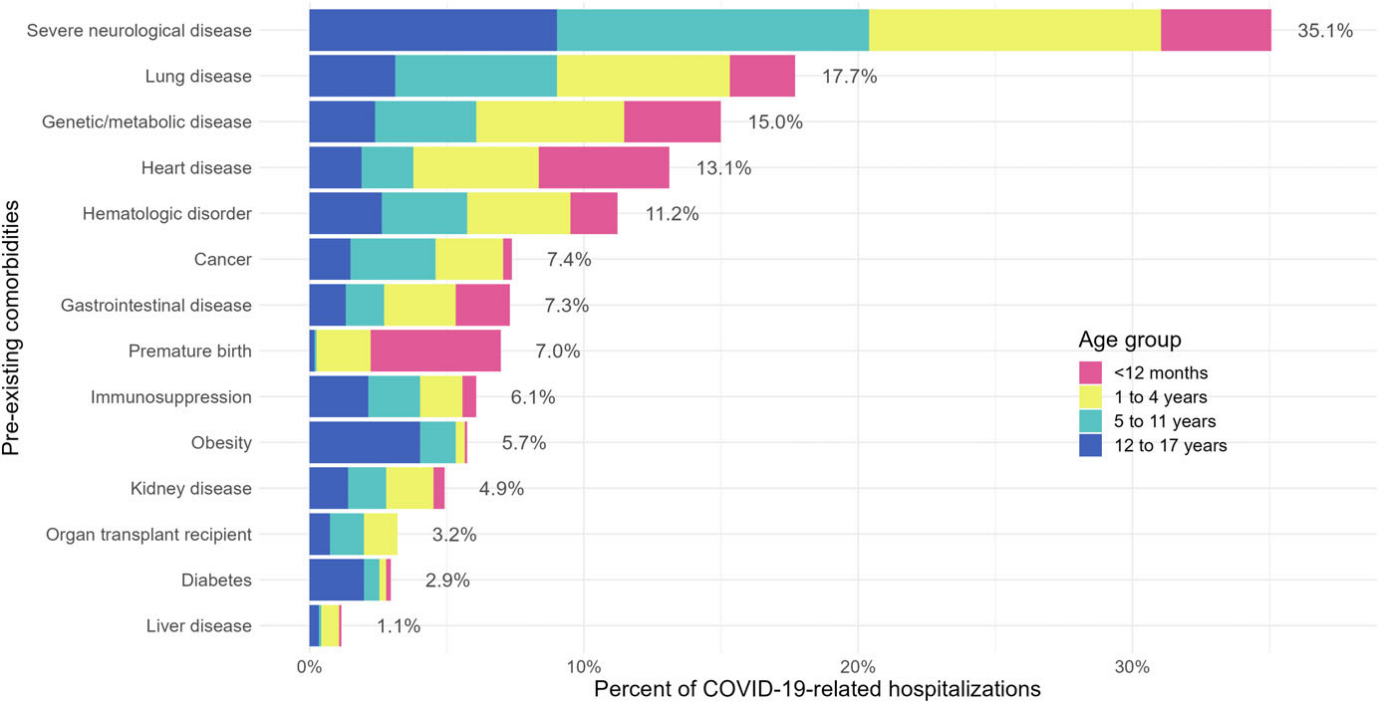
Pre-existing comorbidities among pediatric patients admitted due to COVID-19 by age group.

There was no difference in the proportion of pediatric patients with at least one reported pre-existing comorbidity between the pre-Omicron and Omicron-dominant periods. Among 2,700 patients whose SARS-CoV-2 infection contributed to their admission, 1,221 (45%) had at least one reported pre-existing comorbidity. Of these 1,221 patients, 35% reported severe neurological disease, 18% reported lung disease, and 15% reported genetic/metabolic disease (Figure 2). Among patients younger than 12 months with a pre-existing comorbidity who were admitted due to their SARS-CoV-2 infection (n = 234), the most commonly reported conditions were heart disease and premature birth at 25% each, followed by severe neurological disease (21%). Patients with pre-existing comorbidities in the age groups 1–4 years (n = 390) and 5–11 years (n = 326) reported higher proportions of severe neurological disease (33% and 43%, respectively) and lung disease (20% and 22%, respectively). Severe neurological disease (41%) was also the most commonly reported pre-existing condition in the 12–17-year age group (n = 271). Contrary to the younger age groups among whom obesity (body mass index ≥30 kg/m^2^) was reported infrequently (< 5% of patients with a pre-existing condition), 18% of patients in the 12–17-year age group were reported to have pre-existing obesity.

Of the 5,052 patients whose acquisition source was reported, there were 272 HA-COVID-19 infections (5.4%); 90% occurred during Omicron. Table 2 presents characteristics of pediatric patients with HA- and CA-COVID-19. While there was no difference in age distributions, 63% (166/265) of patients with HA-COVID-19 had a pre-existing comorbidity compared to 44% (1,536/3,469) with CA-COVID-19 (*P* < 0.001). Among those who were eligible for vaccination at the time of positive test, a greater proportion of HA-COVID-19 patients received at least one dose of a COVID-19 vaccine compared to those with CA-COVID-19 (63% vs 47%, *P* < 0.01). A higher proportion of patients with CA infection experienced COVID-19-related ICU admission compared to HA-COVID-19 patients (13% vs. 6%, *P* < 0.01). No differences in deaths (0% (0/272) for HA-COVID-19 vs 1.1% (38/3,541) for CA-COVID-19 patients) and median lengths of stay among discharged patients were observed between HA and CA cases. However, 24% remained in hospital 30 days after a positive test result for HA-COVID-19, compared to 2.1% with CA-COVID-19 (*P* < 0.001).

**Table 2.** Characteristics of pediatric patients hospitalized with COVID-19 by acquisition source, March 2020–December 2022

Characteristic	Healthcare-associated n = 272^a^	Community-associated n = 4,780^a^	*P* value^b^
**Age (years)**			0.44
—Median (IQR)	2.0 (0.0, 13.0)	3.0 (0.0, 11.0)	
—Range	0.0, 17.0	0.0, 17.0	
**Age category**			
—<6 months	55/272 (20%)	1,036/4,780 (22%)	0.57
—6–12 months	30/272 (11%)	409/4,780 (8.6%)	0.16
—1–4 years	65/272 (24%)	1,287/4,780 (27%)	0.27
—5–11 years	43/272 (16%)	959/4,780 (20%)	0.087
—12–17 years	79/272 (29%)	1,089/4,780 (23%)	0.017
**Sex**			0.050
—Male	132/271 (49%)	2,604/4,751 (55%)	
—Female	139/271 (51%)	2,147/4,751 (45%)	
**Wave**			0.001
—Pre-Omicron	28/272 (10%)	859/4,780 (18%)	
—Omicron	244/272 (90%)	3,921/4,780 (82%)	
**Median length of stay**, days (IQR)	3.0 (1.3, 6.0)	3.0 (1.0, 5.0)	0.082
**Number of COVID-19 vaccine doses received** ^c^			
—0	28/76 (37%)	471/882 (53%)	0.006
—1	9/76 (12%)	119/882 (13%)	0.68
—2	35/76 (46%)	261/882 (30%)	0.003
—3+	4/76 (5.3%)	31/882 (3.5%)	0.35
**Pre-existing comorbidity**	166/265 (63%)	1,536/3,469 (44%)	<0.001
**Symptomatic**	216/249 (87%)	3,130/3,465 (90%)	0.067
**Receipt of antiviral**	24/262 (9.2%)	225/3,455 (6.5%)	0.10
**Receipt of steroids**	70/258 (27%)	992/3,421 (29%)	0.52
**ICU admission (all-cause)**	39/271 (14%)	554/3,510 (16%)	0.54
—ICU admission attributable to COVID-19	17/271 (6.3%)	443/3,510 (13%)	0.002
**Mechanical ventilation**	24/269 (8.9%)	249/3,485 (7.1%)	0.28
**ECMO**	0/271 (0%)	7/3,556 (0.2%)	>0.99
**30-day outcome**			
—Patient survived and discharged	187/272 (69%)	3,347/3,541 (95%)	<0.001
—Patient alive, still in hospital	66/272 (24%)	74/3,541 (2.1%)	<0.001
—Patient survived and transferred	19/272 (7.0%)	65/3,541 (1.8%)	<0.001
—Patient died (all-cause)	0/272 (0%)	38/3,541 (1.1%)	0.11
—Death attributable to COVID-19	N/A	28/32 (88%)	>0.99

IQR, interquartile range; ICU, intensive care unit.

a n/N (%).

b Wilcoxon rank sum test; Pearson’s Chi-squared test; Fisher’s exact test.

c Patients ineligible for vaccination at time of positive SARS-CoV-2 test were excluded.

Among these 166 patients with an HA-COVID-19 infection and a pre-existing comorbidity, 31% belonged to the 12–17-year age group, followed by 23% in the 1–4-year age group. Severe neurological disease was most commonly reported (21%), while cancer, chronic heart disease, and lung disease were each reported by approximately 12% of patients.

## Discussion

Among pediatric patients with laboratory-confirmed COVID-19 in Canada, the Omicron-dominant period differed significantly from earlier waves of the pandemic, with a four-fold increase in number of pediatric hospitalizations with COVID-19, higher proportion of admissions due to COVID-19, lower proportion requiring intensive care due to COVID-19, and near-doubling in the proportion of HA infections. There was no difference in the proportion of pediatric patients with at least one reported pre-existing comorbidity between both periods, with neurological disease reported most frequently among patients greater than age 12 months. HA-COVID-19 cases were more likely to have pre-existing comorbidity compared to CA-COVID-19 cases but significantly lower rates of ICU admission attributable to COVID-19.

During the Omicron-dominant period, most hospitalized pediatric patients with COVID-19 within the national network did not experience severe outcomes; over 90% of patients were discharged within 30 days following their infection, and length of stay was shorter compared to the pre-Omicron period (2 days vs 3 days). Although the majority (81%) of pediatric cases were reported during the Omicron-dominant period and the proportion of hospitalizations due to COVID-19 increased, a lower proportion required intensive care and there was no difference in mortality compared to the pre-Omicron period. Interestingly, a higher proportion of CA pediatric patients experienced ICU admission attributable to COVID-19 compared to HA pediatric patients (13% vs 6%) and despite low mortality, 90% of pediatric COVID-19 deaths were attributable to COVID-19. Our findings are in contrast with the adult COVID-19 experience during Omicron, when fewer patients were admitted due to COVID-19.^21,22^ These differences may relate to population-level immunity, particularly among younger age groups, and warrant further study. Our results support other findings that Omicron-related infections, while increasingly transmissible and possibly immune-evasive have led to higher proportions of pediatric CA- and HA-COVID, generally cause milder illness, especially among younger ages and vaccinated populations.^23–26^

Throughout the study period, vaccines became available for the pediatric population, although the rollout was staggered by age group and variable across provinces and territories. In the pre-Omicron period, most hospitalizations were among children and youth not vaccinated against COVID-19. A decrease in the proportion of cases among age groups eligible for COVID-19 vaccination was observed over successive pre-Omicron waves, primarily among individuals aged 12–17 years, in keeping with local and international experience.^25,27,28^ This trend was also noted among children aged 5–11 years, with decreasing proportions of COVID-19-related hospitalizations at the end of the pre-Omicron period when vaccines became available to this age group.^18^ Contrary to the older age groups, the proportion of pediatric cases in the under-5 years age group increased over time and represented greater than 60% of pediatric cases in wave 7. Vaccination was not approved for individuals aged 6 months to 4 years until July 2022, long after public health measures around masking and distancing were more relaxed in communities and school settings. Moreover, vaccine coverage among children under 5 years was variable across provinces and territories, but generally much lower than among older children and youth at only 9% receiving at least one dose by the end of 2022.^29^ These factors may have contributed to the observed higher proportion of unvaccinated, laboratory-confirmed hospitalized cases in the Omicron-dominant period.

In our study, almost half (45%) of patients admitted due to their COVID-19 illness had at least one pre-existing comorbidity; nearly two-thirds with HA infection reported a pre-existing comorbidity; and 79% of 28 patients whose death was attributable to COVID-19 reported a pre-existing comorbidity. These observations, and the higher proportions of patients in our cohort with severe neurologic and lung conditions relative to other pre-existing comorbidities, are consistent with earlier reports of children with medical complexity being at increased risk of severe illness.^30–33^

Severe neurological disease was the most frequently reported pre-existing comorbidity, aligning with a case-control study in Alberta indicating that neurological disease was more common among patients with COVID-19 than those without.^34^ Pre-existing cardiac conditions were frequently reported among younger children (<12 months) and obesity was more frequently reported among older children (12–17 years), agreeing with other studies observing higher rates of severe COVID-19, including worsening heart failure, among children with congenital heart disease as well as obesity.^30,33,35^

The majority (90%) of HA-COVID-19 cases were reported during the Omicron-dominant period, with a greater proportion of patients with HA-COVID-19 having at least one pre-existing comorbidity and longer length of hospital stay compared to CA-COVID-19 patients. Longer length of hospital stay may be attributable to these underlying comorbidities rather than COVID-19 disease severity, as our results indicate that a lower proportion of patients with HA-COVID-19 were admitted to the ICU due to COVID-19-related complications. These results differ from a systematic review and meta-analysis of adults with COVID-19 that found increased risk of mortality among HA cases, particularly among immunocompromised patients.^36^

### Strengths

There are several strengths to this study. The CNISP VRI network is the only national surveillance platform for HA infection surveillance in Canada with data submitted by experienced infection control professionals. As such, the study had a large sample size and rich patient-level data on pediatric CA- and HA-COVID-19 cases from all the tertiary care pediatric hospitals and a broad network of pediatric programs within adult centers across all provinces and one territory during the COVID-19 pandemic. The standardized protocol for the patient questionnaires was co-developed by a working group of subject matter experts in infection prevention and control and iteratively refined to consistently capture detailed data on demographics, source of acquisition, outcome, and treatment. Periodic assessments were performed with the CNISP team to ensure robustness and quality of data over successive pandemic waves.^37^

### Limitations

An important limitation was that the analyses did not account for immunity due to previous infection or effectiveness of new treatments. Moreover, surveillance data have inherent limitations, including under-ascertainment and/or underreporting, lack of timely reporting, and lack of completeness of every variable in the questionnaire. Minor changes were made to the COVID-19 case definition over time to provide clarifications and room for best clinical judgment. Unless clearly stated by the medical team, there may have been inconsistencies in attributing COVID-19 illness as the reason for admission versus an incidental finding.^38,39^ Furthermore, the analyses provided an overview of pediatric COVID-19 in Canada but did not compare across jurisdictions, nor jurisdictional differences in COVID-19-related public health measures.

We demonstrate that pediatric COVID-19 cases in Canada increased following the emergence of the Omicron variant; however, disease severity remained low with more than 90% survival. Pre-existing health conditions were common among pediatric patients hospitalized due to their COVID-19 illness, with the most common conditions being severe neurological disease followed by lung disease, particularly among children aged less than 5 years. HA-COVID-19 significantly increased during the Omicron-dominant period, underscoring the importance of organizational infection prevention and control policies to support staff and caregiver adherence to routine practices and additional precautions. Further investigation into regional differences (e.g., vaccination roll out, public health and hospital-based measures, population differences) could provide additional insight into the findings and potentially identify other factors that influenced COVID-19 clinical patterns in Canadian pediatric populations.

## Research transparency and reproducibility

The surveillance protocol is available online. Data-sharing requests will be considered and reviewed by the Public Health Agency of Canada and individual site investigators.
